# A Case of Subacute Combined Degeneration Secondary to Recreational Whippet Use

**DOI:** 10.7759/cureus.44203

**Published:** 2023-08-27

**Authors:** Jarrett Rong, Jonathan Martinez, Alexis Aiman, Joshua Stadler

**Affiliations:** 1 Internal Medicine, The University of Tennessee Health Science Center School of Medicine, Memphis, USA; 2 Internal Medicine Residency, University of Pittsburgh Medical Center, Harrisburg, USA; 3 Internal Medicine, UPMC (University of Pittsburgh Medical Center) Community Osteopathic, Harrisburg, USA

**Keywords:** general internal medicine, subacute combined degeneration of the spinal cord, causes of vitamin b12 deficiency, nitrous oxide abuse, nitrous oxide inhalation

## Abstract

‘Whippets’ or nitrous oxide (N_2_O) abuse is a rare etiology of B12 deficiency and subacute combined degeneration (SCD). Often used in the medical field as an anesthetic, recreational use has rapidly increased given its euphoric effects. Easy accessibility over the counter at local stores due to the fact that it has bacteriostatic effects useful for canisters of creams and perishable goods. This makes N_2_O, or “laughing gas,” easy to obtain. Long-standing abuse of N_2_O can lead to deleterious effects on the central nervous system, including SCD, polyneuropathy, and death. Presentation includes frequent falls, ataxic gait, weakness in the lower extremities, and neuropathy. Herein, we present a case of a 25-year-old male with no past medical history presenting with SCD in the setting of longstanding recreational whippet use. Our case highlights an important consideration for all specialties, including emergency medicine, psychiatry, family medicine, and internal medicine physicians.

## Introduction

Subacute combined degeneration (SCD) of the spinal cord is a neurodegenerative pathology, consisting of the degeneration of the dorsal and lateral white matter of the spinal cord. The disease is typically caused by long-standing vitamin B12 deficiency. The degeneration causes progressive weakness, sensory ataxia, and paresthesias. Left untreated, this disease will lead to spasticity, paraplegia, and incontinence. Supplemental vitamin B12 replacement typically halts the progression of the disease and improves neurological symptoms, but is not always curative. Vitamin B12 deficiency secondary to nitrous oxide (N_2_O) use is a rare cause of SCD. SCD is not typically or immediately life-threatening, but if left untreated, it can cause longstanding debility. Unfortunately, as stated above, some patients may suffer from debilitating extremity weakness or long-standing symptoms of polyneuropathy [1]. Vitamin B12 is a required cofactor in the synthesis of methionine. Adequate development of oligodendrocytes and myelin sheath requires methionine. The pathogenesis of SCD in the setting of N_2_O use is secondary to the irreversible oxidation of vitamin B12 [2]. This irreversible oxidation of vitamin B12 renders methionine synthesis to a halt, unable to produce phosphatidylcholine and sphingomyelin - cellular components required to create the myelin sheath. SCD secondary to N_2_O use is increasingly more common among young adults [1], likely due to ease of access.

Common presentations include lower extremity weakness, disruption in vibratory sensation, and gait abnormalities, although there have been documented cases of neuropsychiatric manifestations as well [1-3]. B12 deficiency has multiple etiologies, thus competing diagnoses need to be ruled out to make a confident diagnosis with a careful review of social history, imaging, and laboratory data. Common causes include poor oral intake, often in the setting of vegetarian and vegan diets, malabsorption, history of sleeve and gastrectomy surgeries, intrinsic factor deficiency, and celiac disease. A rare and often forgotten etiology of underlying B12 deficiency includes ‘whippet’ or N_2_O use. We present the case of a 25-year-old male with SCD secondary to N_2_O use. He was hospitalized for further evaluation and treatment of profound lower extremity weakness and was subsequently found to have SCD secondary to N_2_O.

## Case presentation

**Figure 1:**
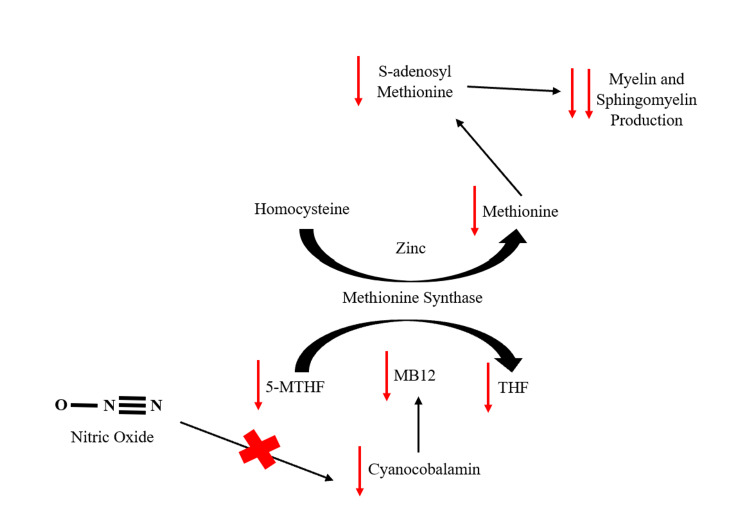
The irreversible oxidation of B12 via nitric oxide and its effects on myelin activity 5-Methyltetrahydrofolate (5-MTHF), Methyl-B12 (MB12), Tetrahydrofolate (THF)

Figure 1 displays the irreversible oxidation of vitamin B12 by N_2_O.

**Figure 2:**
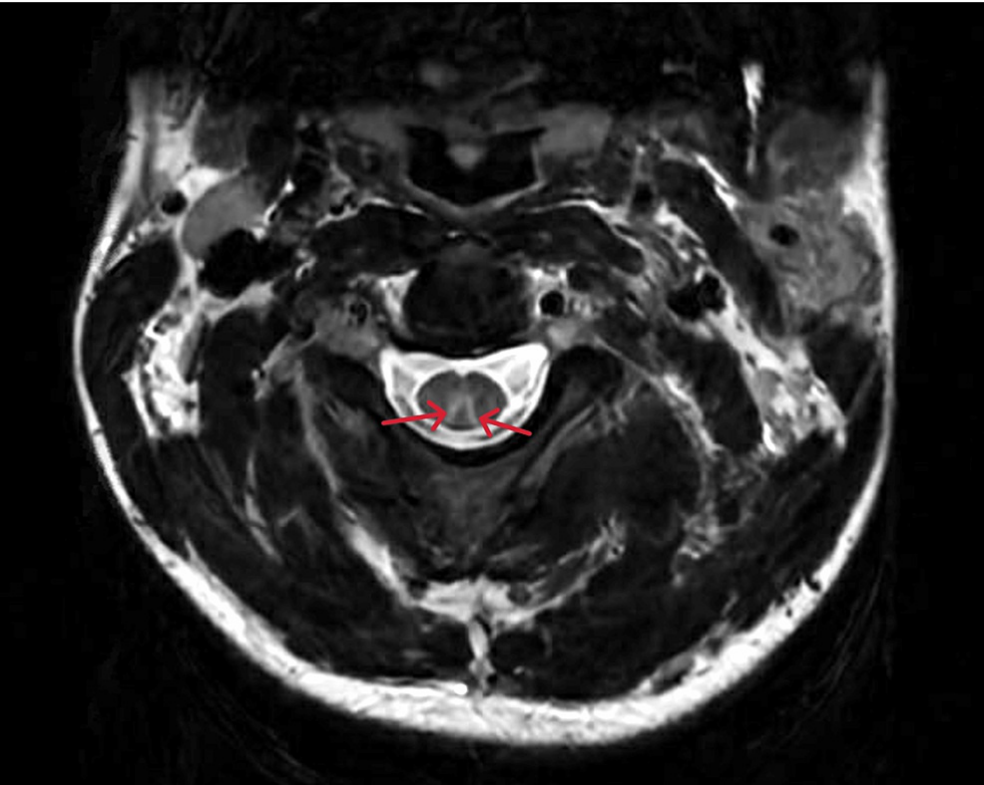
Axial MRI of the cervical spine demonstrating a T2 symmetric bilateral high intensity in the dorsal columns in the form of an inverted ‘V’ Red arrows point to the high-intensity regions.

**Figure 3:**
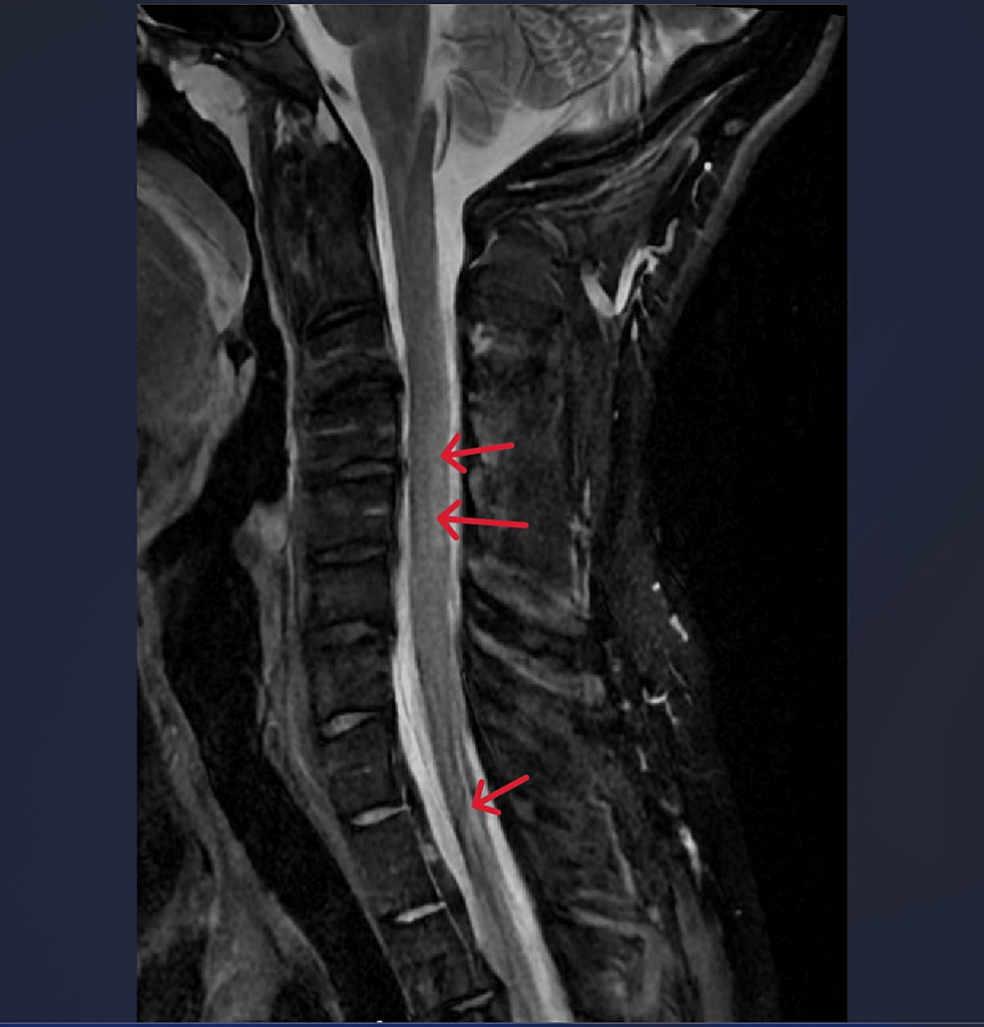
Sagittal MRI cervical spine demonstrating a T2 STIR hyperintense intramedullary signal extending from the level of mid-C2 to the inferior margin of the C7 vertebral body Red arrows point to hyperlucent areas indicative of neurodegeneration.

This case describes a 25-year-old, previously healthy male who presented to the hospital with a three-week history of weakness and numbness of the bilateral lower extremities. The patient's symptoms were progressively worsening. No known provoking or alleviating factors were present. He had no previous medical history and took no medications. Social history included recreational N_2_O and cannabis use with a diet that consisted primarily of fast food and red meat. Vitals signs were within normal limits on admission. The physical examination was significant for ataxic gait, positive Romberg test, 1/5 strength in bilateral plantar and dorsiflexion, reduced proprioception, hyperreflexia, and preserved pain and temperature sensation. Upper extremity strength revealed: 4/5 bilateral upper extremity strength with bilateral reduced grip strength 4/5. The pertinent laboratory work-up can be found in Table 1. These findings included macrocytic anemia with severely decreased B12 serum level and normal serum folate level. Initial differential diagnoses included vitamin B12 deficiency secondary to recreational N_2_O use, vitamin B12 deficiency secondary to intrinsic factor deficiency, syphilis, and HIV myelopathy. The patient denied the use of metformin or proton pump inhibitors. Brain magnetic resonance imaging (MRI) was normal, but MRI spine showed hyperintense lesions involving the dorsal columns in the form of an inverted 'V', extending from the cervical spine into the upper thoracic spine, suggestive of SCD as seen in Figure 2 and Figure 3. The medical team performed further deliberation and attempted to address other potential causes of vitamin B12 deficiency. Unfortunately, we were not able to perform H. pylori testing, intrinsic factor testing for pernicious anemia, or aquaporin-4 antibody-positive neuromyelitis due to constraints with medical resources but neurology was consulted and agreed with the medicine team's reasoning for the diagnosis of SCD from chronic whippet abuse, given the culmination of social history significant for whippet use, MRI findings suggestive of SCD, and decreased levels of serum vitamin B12. Of note, this patient did not present with gastrointestinal symptoms suggestive of pernicious anemia or gastritis and did not carry a diagnosis of diabetes nor take diabetic medications or proton pump inhibitors. The patient was started on intramuscular vitamin B12 1,000 mcg daily. Hospitalization was uneventful, and the patient was compliant with intramuscular vitamin B12 administration daily for the duration of two days, in addition to acute physical therapy sessions with minimal improvement. He was encouraged by our internal medicine team and physical therapy team to go to inpatient rehabilitation, but he declined, with plans to return to his home. Treatment guidelines consisted of intramuscular vitamin B12 1000 mcg, once per week for a duration of four weeks, with plans to transition to oral supplementation with B12 daily [4]. The patient repeatedly requested to go home, and with shared decision-making with the patient, we elected for two days of intramuscular B12 followed by oral supplementation, with plans for close follow-up outpatient. He was discharged on oral 1000 mcg of vitamin B12 supplementation daily and a rolling walker and an outpatient follow-up was scheduled for the following week. Despite multiple phone call attempts to counsel on addiction, the patient was lost to follow-up.

**Table 1:** Laboratory work-up on admission *grams per deciliter (g/dL), femtoliters (fL), pictograms per milliliter (pg/mL), micrograms per liter (mcg/L)

Laboratory Value	Value on Admission	Normal Value
Hemoglobin (Hgb)	12.7 (g/dL)	14.8 – 18.0 (g/dL)
Hematocrit (Hct)	37.8 %	40 – 50 %
Mean corpuscular volume (MCV)	102.5 (fL)	80 – 100 (fL)
Rapid plasma reagin (RPR)	Non-reactive	Non-reactive
HIV 1 & 2	Negative	Negative
Vitamin B12	119 (pg/mL)	180 – 914 (pg/mL)
Folate	23.24 (mcg/L)	3 – 20 (mcg/L)

## Discussion

Vitamin B12, or cyanocobalamin, is a water-soluble vitamin that is not naturally synthesized by the human body and must be gained from foods such as meat and dairy products. Once consumed, vitamin B12 in the stomach is bound by intrinsic factors, produced by parietal cells of the stomach, promoting the transport and absorption of the vitamin in the ileum. Within the body, vitamin B12 plays a crucial role in maintaining the integrity of the nervous system. It acts as a cofactor in the synthesis of methionine. Methionine then goes on to support the production of phosphatidylcholine and sphingomyelin, which are major components of myelin. In addition, vitamin B12 plays a role in DNA synthesis, which is crucial in the maintenance of the myelin-producing oligodendrocytes.

Disruption of any of the processes can lead to vitamin B12 deficiency and manifest as SCD due to loss of support for the central nervous system. Individuals adhering to a strict plant-based diet may experience vitamin B12 deficiency as plant-based foods do not contain vitamin B12. Pernicious anemia or autoimmune anemia is another common cause of vitamin B12 deficiency. This process involves autoimmune destruction of the parietal cells, preventing intrinsic factors from binding to and facilitating the absorption of vitamin B12. Although uncommon, N_2_O can be a cause of SCD in an otherwise healthy young man. N_2_O, via oxidation, irreversibly inactivates vitamin B12 and can lead to symptoms of SCD [2]. Although our team did not obtain laboratory work-up specific for intrinsic factors, this should be a strong consideration in a patient with poor absorption and lower extremity weakness. Given the social history, decreased B12 levels, MRI findings, expertise of our Neurology colleagues, and efficacy of hospital resources, we suggest the cause of lower extremity weakness in this patient was due to SCD secondary to N_2_O use.

To date, there are primarily case studies of SCD secondary to nitric oxide use, so the incidence is likely underrepresented in the literature at large. However, Li and colleagues found that the incidence of neurologic manifestations in 61 patients ages 15-30 with SCD secondary to nitric oxide abuse was distal limb numbness [5]. Vulnerable populations include those abusing nitric oxide as a recreational drug, exposure during anesthesia, and those with a recent history of scuba diving and decompression sickness. Overt B12 deficiency affects spinal tracts within the spinal column causing SCD. The posterior columns are primarily affected leading to signs and symptoms of aberrant vibration and position sensation, lower extremity weakness, hyperreflexia, and gait abnormalities. In severe cases, bowel incontinence and even death have been observed. We observed profound functional deficits including, ataxic gait, lower extremity weakness, and hyperreflexia in our patient. In addition to labs and physical examination, surgical history, medication history, social history, and MRI are critical to support the diagnosis of N_2_O-induced vitamin B12 deficiency. Our patient had no history of bariatric surgery, celiac disease, Crohn’s disease, histamine H2 receptor antagonist use, or metformin use, which are common culprits of vitamin B12 deficiency [6,7]. The patient also had a diet consisting predominantly of red meats, which likely rules out nutritional deficiency. On MRI of the spine, our patient demonstrated symmetric involvement on T2 weighted imaging showing high signal intensity lesions spanning multiple segments of the spinal cord within the posterior columns of the spinal cord, sparing the anterior columns as shown in Figure 2. Additional findings included the “inverted V sign,” representing a lesion on the dorsal column of the spinal cord seen in an axial MRI of the spine associated with SCD [8]. The patient’s history of N_2_O abuse, MRI findings, and vitamin B12 deficiency strongly suggest N_2_O-induced SCD.

Oftentimes, N_2_O-induced SCD lasting less than three months can be reversed. Treatment guidelines consist of intramuscular vitamin B12 1000 mcg, once per week for a duration of four weeks, with plans to transition to oral supplementation with B12 daily [4]. However, it must be noted that patients with known malabsorption or a history of bariatric surgery would benefit from parenteral administration of vitamin B12. Response to treatment varies per individual, and symptomatic improvement can occur within two weeks. Symptoms, however, may take months to a year to completely resolve [9]. In some cases of prolonged SCD, there may be irreversible neurological sequelae of the disease. Lastly, vitamin B12 compliance and SCD symptoms should be closely monitored in the outpatient setting, as patients should be counseled on the adverse effects of N_2_O with emphasis on addiction counseling.

## Conclusions

In summary, our patient had a rare etiology of bilateral lower extremity and upper extremity weakness secondary to recreational N_2_O use. Profound symptomatic B12 deficiency secondary to recreational N_2_O use is a rare consideration among internal medicine physicians. Clinical suspicion for SCD secondary to N_2_O should be considered in patients who abuse drugs, have prior anesthetic exposure, or those with a career in or recent scuba diving history. A thorough social history and systematic evaluation are necessary to rule out other causes of bilateral lower extremity and upper extremity weakness in a young male. In addition to iron studies and vitamin studies, MRI should be performed to evaluate pathologic findings consistent with SCD. B12 deficiency secondary to N_2_O use should be treated early with aggressive vitamin B12 replacement in the setting of functional recovery. Addiction counseling should be offered upon discharge for optimal long-term abstinence.
